# Simultaneous Determination of Preservatives in Dairy Products by HPLC and Chemometric Analysis

**DOI:** 10.1155/2017/3084359

**Published:** 2017-04-03

**Authors:** Fatemeh Zamani Mazdeh, Sima Sasanfar, Anita Chalipour, Elham Pirhadi, Ghazal Yahyapour, Armin Mohammadi, Akram Rostami, Mohsen Amini, Mannan Hajimahmoodi

**Affiliations:** ^1^Pharmaceutical Quality Assurance Research Center, Faculty of Pharmacy, Tehran University of Medical Sciences, Tehran, Iran; ^2^Food and Drug Administration, Tehran University of Medical Sciences, Tehran, Iran; ^3^Drug and Food Control Department, Faculty of Pharmacy, Tehran University of Medical Sciences, Tehran, Iran; ^4^Medical Chemistry Department, Faculty of Pharmacy, Tehran University of Medical Sciences, Tehran, Iran

## Abstract

Cheese and yogurt are two kinds of nutritious dairy products that are used worldwide. The major preservatives in dairy products are sodium benzoate, potassium sorbate, and natamycin. The maximum permitted levels for these additives in cheese and yogurt are established according to Iranian national standards. In this study, we developed a method to detect these preservatives in dairy products by reversed phase chromatography with UV detection in 220 nm, simultaneously. This method was performed on C_18_ column with ammonium acetate buffer (pH = 5) and acetonitrile (73 : 27 v/v) as mobile phase. The method was carried out on 195 samples in 5 kinds of commercial cheeses and yogurts. The results demonstrated insufficient separation where limit of detection (LOD) and limit of quantitation (LOQ) ranged from 0.326 to 0.520 mg/kg and 0.989 to 1.575 mg/kg in benzoate and sorbate, respectively. The correlation coefficient of each calibration curve was mostly higher than 0.997. All samples contained sodium benzoate in various ranges. Natamycin and sorbate were detected in a remarkable amount of samples, while, according to Iranian national standard, only sorbate is permitted to be added in processed cheeses as a preservative. In order to control the quality of dairy products, determination of preservatives is necessary.

## 1. Introduction

Nowadays, preservation techniques have considerable role in food industry. Generally, they are used for improving the quality with boosting durability of products and thus enhancing food shelf life. The rate of food spoilage can be controlled by many procedures such as suitable packaging for preventing available oxygen, sterilization, pasteurization, dehydration (drying), smoking, freezing, and food additive [1]. One of the methods of preservation in dairy products is consumption of additives. According to codex alimentarius commission, these substances are added to food for maintaining or improving nutritional quality [2]. In late 1988, the European Community adopted the laws for use of additives in foodstuffs for human consumption [3]. In this regard, the Institute of Standard and Industrial Research of Iran (ISIRI) regulated the limitation of consumption food additives such as preservative.

Dairy products as an important nutritional recommended consuming in daily and appropriate amounts. The safety of dairy products should be considered in the presence of preservatives. The acceptable daily intake (ADI) represents amount of daily consumption of substance without any risk even for a lifetime. According to ADI, the maximum permitted limit for food additives is based on mg/kg of body weight. The most commonly used preservatives in dairy products such as cheese and yogurt are benzoate, sorbate, and natamycin [4]. These compounds are generally used to inhibit various types of microorganisms (e.g., bacteria, yeasts, and molds).

Sodium benzoate (E211) is known as the first chemical preservative approved in food products by the US Food and Drug Administration (FDA). Its solubility is more than other salt of benzoate like potassium and calcium. In an extensive review Sieber et al. (1995) investigated natural occurrence of benzoic acid in many types of dairy products [5]. Researches demonstrated that sodium benzoate is affected by pH. Hence, increasing the pH of a medium decreased the effectiveness of benzoate. It is active in acidic condition against bacteria, yeast, and fungi [6].

Potassium sorbate (E202) is produced from the potassium salt of 2,4-hexadienoicacid (sorbic acid). The carboxyl group and conjugated double bonds in the sorbate structure are reactive and can have strong effect on the antimicrobial activity as well as the quality and the safety of the product [7]. It is widely applied in food industry as preservative because of high stability, solubility, and being easy to use. The combination of sorbate and benzoate has more effective role than uses of each one in cheese lonely, for preventing and delaying growth of mold along with propionic acid [8–11]. Natamycin (E 235) is a tetraene polyene macrolide and has amphoteric structure like other macrolides. Low solubility in water (approximately 40 mg/kg) is an advantage for natamycin. Especially in surface treatment of food like cheese, the mold grows on the surface of cheese which is the major factor limiting the shelf life. Therefore, preservative remains on the surface of product where it is required. Natamycin is active against nearly all yeasts and molds but is not bactericidal [12–14].

The use of benzoate, sorbate, and natamycin in dairy products as additives is forbidden in Iran; only sorbate is permitted for additives in processed cheeses at a maximum level of 1000 mg/kg. Therefore, determination of these preservatives is needed for safety and quality control of dairy products.

There are various analytical methods for determination of these preservatives in cheese and yogurt such as thin layer chromatography, UV spectroscopy, high performance liquid chromatography (HPLC), and gas chromatography (GC). HPLC is the most common analytical technique for the detection and quantification of these preservatives. A practical method should be developed to identify all preservatives simultaneously [15, 16].

The aim of the present study is to develop effective, simple, efficient, and fast RP-HPLC method to detect the three preservatives in one runtime. A simple extraction procedure was selected for measuring three preservatives from cheese and yogurt matrix. The result of the validation method parameters indicates the accuracy of the method. Finally, in order to ensure effective quality control in dairy products, the method was tested on 195 samples of commercial cheeses and yogurts in Iranian markets.

## 2. Methods and Materials

### 2.1. Sampling

In this study, 195 samples of 15 brands and 5 types from commercial cheeses and yogurts were purchased from supermarkets located in Tehran. The cheeses samples were categorized as follows: cream, processed, Labneh (Iranian cheese), lactic, and brined cheeses, and yogurts sample were classified as follows: common yogurt and probiotic and flavored yogurt (fruit, vegetable, and strained yogurt) according to Iranian national standard (ISIRI).

### 2.2. Chemicals and Reagents

**Figure 1:**
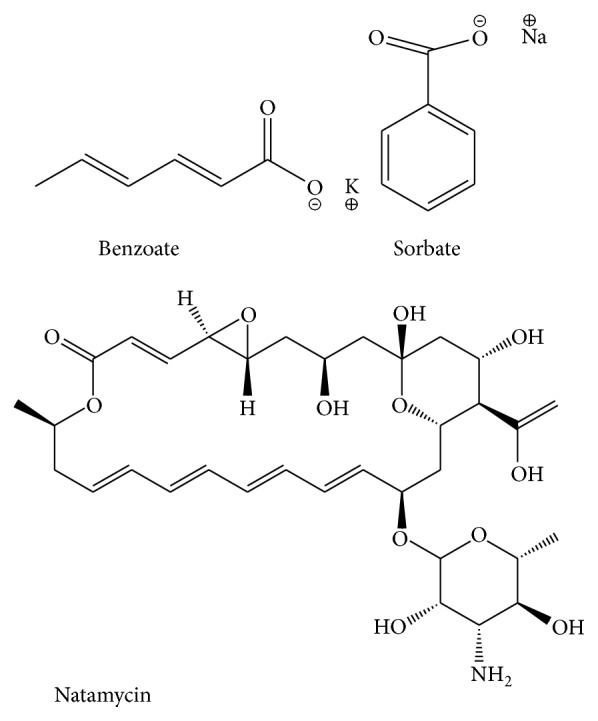
Chemical structures of sodium benzoate, potassium sorbate, and natamycin.

Deionized water was prepared through the Thermo Scientific Barnstead Easy pure II system. Sodium benzoate and potassium sorbate used in this study were obtained from Merck (Darmstadt, Germany). High purity standard of natamycin was purchased from Sigma-Aldrich (Wisconsin, USA). The structures of these preservatives are illustrated in Figure 1.

### 2.3. Determination of Preservatives

#### 2.3.1. Chromatographic Conditions

Chromatographic analysis was carried out on 1200 series HPLC from Agilent technology equipped with four solvent lines, high pressure pump, degasser, 20 *μ*L loop injector, and Ultraviolet detector (UV). The stationary phase is ZORBAX Eclipse-XDB C18 (150 × 4.6 mm, 5 *μ*m) and the mobile phase consisted of an aqueous ammonium acetate buffer (pH = 5) and acetonitrile (73 : 27 v/v). The separation was performed with a flow rate of 1 mL/min and 20 *μ*L injection volume and by setting the UV detector at 220 nm. The chromatographic system was initially conditioned by the mobile phase for minimum of 1 hour.

#### 2.3.2. Preparation of Standards and Samples

Individual stock standard solutions of sodium benzoate and potassium sorbate were prepared in deionized water (1000 mg/kg) and natamycin stock standard was in methanol (1000 mg/kg). The mixed standard solutions were prepared by diluting the stock solutions appropriately with mobile phase to give a concentration between 5 mg/kg and 40 mg/kg.

The pH of samples was measured by 827 model of Metrohm pH meter according to Iranian national standard (ISIRI) [17]. A mass of 10 g sample was weighted in a beaker and diluted with 35 mL solution of acetic acid 1% and methanol (35 : 65 v/v) and then shaken for 15–30 min on stirrer. Then, all content of beaker was transferred to the 50 mL volumetric flask and diluted with extraction solution. Finally, the prepared solution was filtered with 0.45 *μ*m PVDF syringe filter and was injected to HPLC.

#### 2.3.3. Method Validation

The calibration curves of each preservative were prepared over the range of 5–40 mg/kg. The linearity and the correlation coefficient for each standard curve of benzoate, sorbate, and natamycin were calculated.

The detection limit (LOD) and quantitation limit (LOQ) were expressed as follows: DL = 3.3*S*_*b*_/*S* and QL = 10*S*_*b*_/*S*, where *S*_*b*_ was the standard deviation of the response and *S* was the slope of the calibration curve. The estimate of *S*_*b*_ was calculated by using the standard deviation of blank. In order to verify the feasibility of the method, sample recovery was used by analyzing samples before and after the addition of known quantities of benzoate, sorbate, and natamycin. To estimate recovery and the intraday precision (as RSD_r_), each sample was extracted three times as described in “Sample Preparation” section and each extracted sample was analyzed three times in the same day.

### 2.4. Statistical Analysis

The obtained data was analyzed by MATLAB R2010b and the SPSS statistical package, version 16 (SPSS Inc., Chicago, IL, USA). Analysis of variance (ANOVA) was utilized to evaluate the differences of distribution among 5 types of cheeses and yogurts. Statistical significance was set at *p* < 0.05.

## 3. Results and Discussion

195 commercial dairy products from 15 brands in 5 kinds of cheeses (cream, processed, Labneh, lactic, and brined cheeses) and yogurts (regular, probiotic, fruit, vegetable, and flavored strained yogurt) were prepared in April 2015 till August 2015.

In this study, samples were classified for checking with their relevant standards according to the name labeled on the packaging.

### 3.1. Analytical Method

The separate methods for determination of benzoate, sorbate, and natamycin have been used in Iranian national standard [18–22]; but there is no analytical method for determination of three preservatives simultaneously. In this work we intended to develop a rapid, sensitive, and simple method for simultaneous determination of benzoate, sorbate, and natamycin in dairy products such as cheese and yogurt.

To optimize the extraction, the chemical structures of these preservatives were considered (Figure 1). The benzoate and sorbate were completely soluble in water while natamycin had low solubility in water. Therefore, appropriate extraction of benzoate and sorbate along with natamycin was achieved with acetic acid 1% and methanol (35 : 65 v/v) that confirmed a good recovery of three preservatives simultaneously (Table 1).

**Figure 2:**
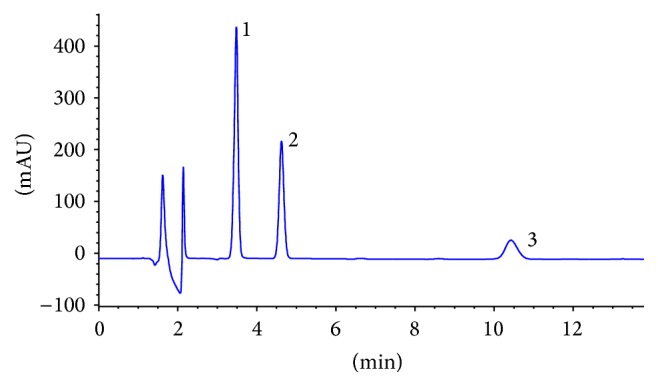
HPLC chromatogram: (1) sodium benzoate, (2) potassium sorbate, and (3) natamycin.

There were other studies for determination of these preservatives individually by chromatographic methods [14]. Some articles reported gradient elution for separation of these at the same time [23, 24]. However, in this study a suitable resolution of reversed phase chromatography was achieved with isocratic elution consisting of ammonium acetate buffer (pH = 5) and acetonitrile (73 : 27 v/v). The separation was carried out within 12 min. These preservatives had different absorption spectra and the maximums of absorption were affected by pH of extraction solvents. For working at a single wavelength, 220 nm was the best. The chromatogram of a standard solution is shown in Figure 2.

Peak identification was achieved by comparing the retention times of standard compounds, and quantification was based on using the calibration curves fitted by linear regression analysis. The results of the calibration data, LOD, LOQ, and the recovery of each preservative are illustrated in Table 1. The recoveries ranged between 82 and 93%, representing that the method had acceptable accuracy and was suitable for simultaneous determination of three preservatives.

In this study, an HPLC method was developed and characterized with minimizing sample preparation for the determination of preservatives in dairy products. There is some similarity between our report and other works, but shorter run-time and suitable resolution of this study can reduce the cost of analysis and have some benefits in quantification as well as LOD, LOQ, and recovery.

### 3.2. Food Samples Analysis

The use of benzoate, sorbate, and natamycin as additive for cheeses and yogurts is forbidden by Iranian national standards [20, 25, 26]; only sorbate is permitted to be added in processed cheeses at a maximum level of 1000 mg/kg. Therefore, for quality control of these dairy products, determination of preservatives is necessary.

The method was applied to several kinds of commercial cheeses and yogurts. Each sample was divided according to kinds and brands, as has been described in sampling and results are shown in Table 2.

**Figure 3:**
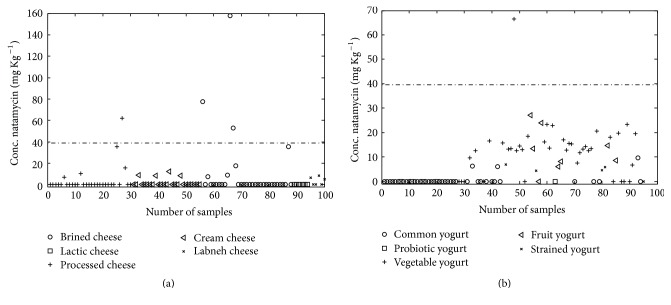
Illustration the natamycin content in (a) cheese and (b) yogurt samples.

45 yogurts and 19 cheeses among 195 samples contained natamycin. Concentrations of natamycin ranged from 4.355 to 66.462 mg/kg in yogurts and 5.249 to 158.025 mg/kg in cheeses. Natamycin was only detected in flavored yogurt (fruit, vegetable, and flavored strained yogurt). It seems that, for reducing the microbial contamination caused by the addition of flavorings, the manufacture added natamycin in flavored yogurt. The amount of natamycin in 21.05% of contaminated cheeses was found in higher concentration than the maximum legal limit of USA FDO (40 mg/kg) [27]. The highest concentration of natamycin was found in cheese samples with high percent of fat; this could be due to a greater solubility of natamycin in this type of cheese. The results of the analysis of natamycin content for the yoghurts and cheeses samples are illustrated in Figure 3. In the same study natamycin was determined in Turkish yoghurt and natamycin was detected in all yoghurt samples and it is not acceptable according to Turkish codex [14].

The results show the presence of benzoate in most of yogurts ranging from 2.076 to 58.188 mg/kg, while benzoate was not detected in two probiotic samples and the lower amount of benzoate in other probiotic yogurts was observed. Therefore, it requires more studies on the probiotic yogurt.

**Figure 4:**
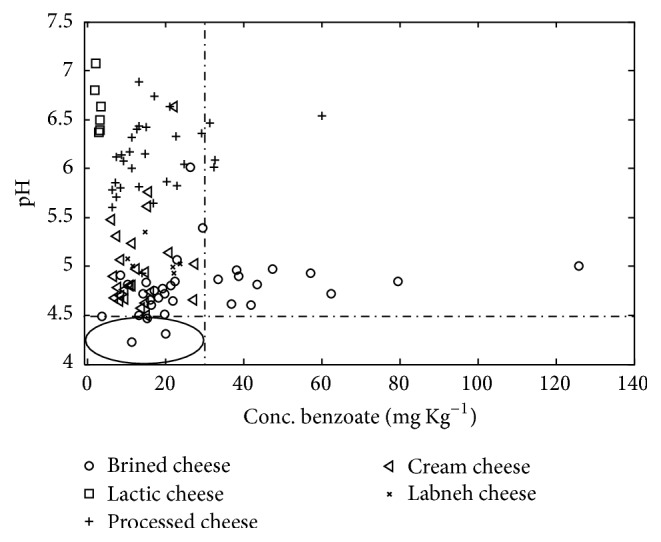
Illustration of benzoate content and the pH in each cheese sample. The samples that located in the violet ellipse have the standard concentration and pH level.

**Figure 5:**
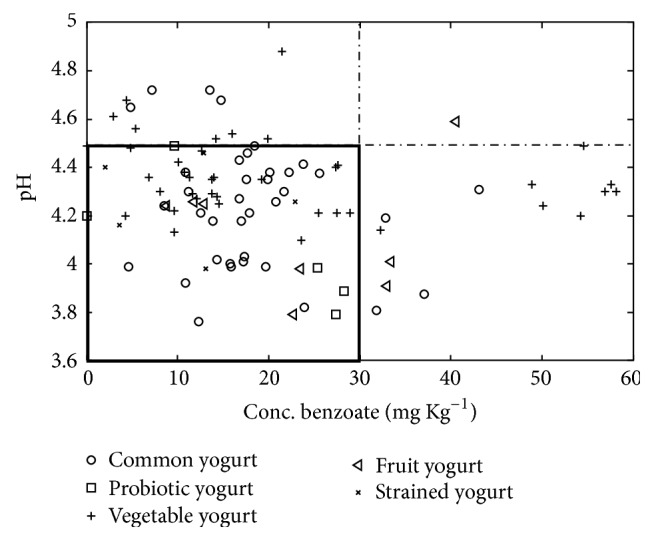
Illustration of the benzoate content and pH in each yogurt sample. The violet rectangle shows the permitted range for concentration of benzoate and the suitable pH for the activation of it.

All of the cheese samples contained benzoate ranging from 1.735 to 125.771 mg/kg, while they had not mentioned adding benzoate on the product labels. In other similar studies it was reported that the concentration of benzoate in Iranian dairy products was lower than 30 mg/kg [23]. In this study benzoate was found higher than 30 mg/kg only in 19 of cheese samples. In Figures 4 and 5 the allowed concentration of benzoate and the pH for each cheese and yogurt sample, respectively, are illustrated. According to the other studies benzoate was not recommended as a preservative at pH ranges higher than 4.5. As shown in Figure 4, in most of the cheese samples, pH of samples was not in the range of effectiveness of benzoate as a preservative [28, 29].

Sieber et al. analyzed benzoate in many kinds of dairy products and their report showed that benzoic acid occurred naturally by microbial degradation or addition of an herbal essence which contains benzaldehyde [5]. Another hand converting hippuric acid to benzoic acid by lactic acid bacteria could be explained as natural occurrence of benzoate milk products [30].

In Iranian national standards, benzoate is not considered as a food additive for dairy products. It seems that a maximum permissible quantity of this preservative should be defined for cheese and yogurt.

**Figure 6:**
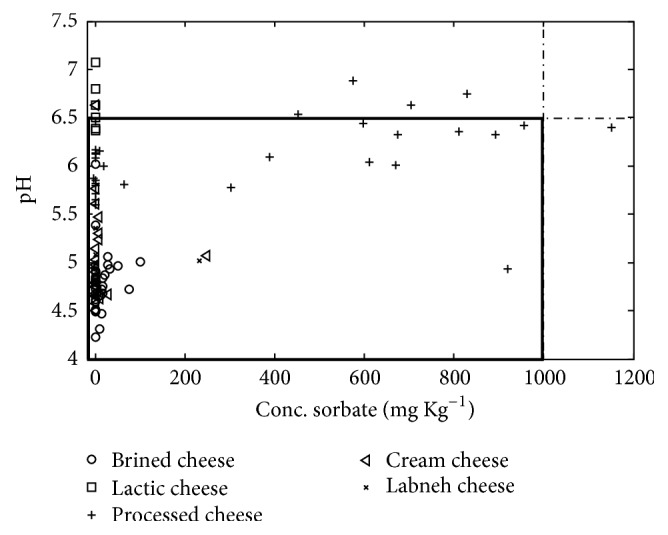
Illustration of the concentration of Sorbate and pH for each cheese sample. The samples that are located outside the violet rectangle have more than allowed concentration of sorbate and pH.

**Figure 7:**
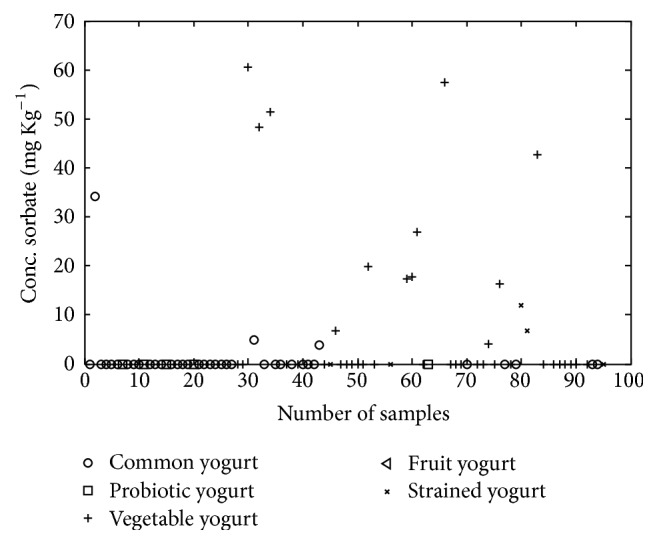
Illustration of concentration content of sorbate in the yogurt samples.

The concentration of sorbate in yogurts and cheeses ranged between 3.809–240.600 and 4.190–1150.320 mg/kg, respectively. 27 yogurt samples and 41 cheese samples were containing sorbate (Figures 6 and 7). According to one-way ANOVA analysis, there was difference between the sorbate concentrations in various kinds of cheeses and yogurts. Tukey analysis showed that flavored yogurt and processed cheese had significant difference with other kinds of yogurt and cheese.

It seems that adding preservatives in flavored yogurt was common as it was seen about natamycin and preservative was added for microbial growth control. Sorbate which was detected in processed cheese could be explained with maximum legal limit in Iran (1000 mg/kg).

**Figure 8:**
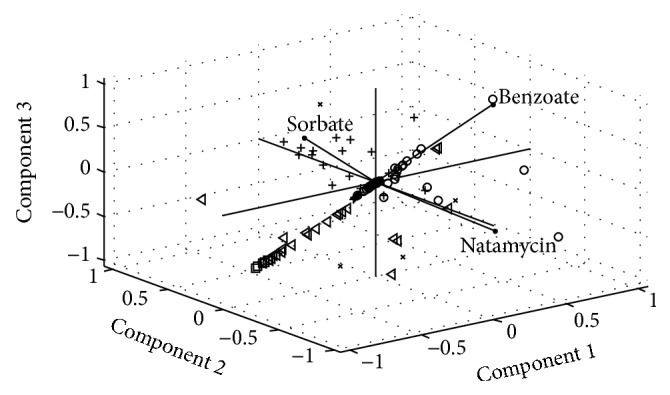
Biplot of first three principal components for the cheese data.

**Figure 9:**
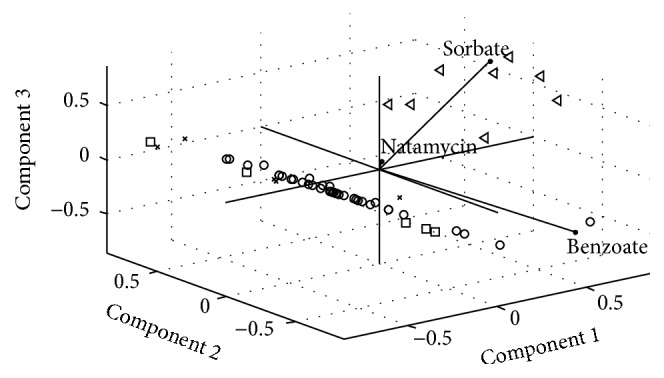
Biplot of first three principal components for the yogurt data.

All achieved results in this study are shown in the abstract space of data that is obtained by applying the principle component analysis. In Figures 8 and 9, the biplot of cheese and yogurt data was shown.

As is clear in Figure 8, lactic cheese that is shown by red circles has the minimum content of preservatives, brined cheese had the maximum amount of benzoate, and processed cheese had the maximum amount of sorbate. In this figure, the different types of cheeses could be classified based on the contents of preservatives

The same analysis was done for the yogurt data. As is shown in Figure 9, vegetable and fruits yogurts had the maximum content of preservatives. In the probiotic yogurt there were not any preservatives. Similar to the cheese samples, the different types of yogurts could be classified based on the contents of preservatives.

## 4. Conclusion

In this research we aim to extract and evaluate the amount of preservatives including sodium benzoate, potassium sorbate, and natamycin among different cheeses and yogurts. Proposed method was demonstrating RP-HPLC method due to its simplicity, reliability, sensitivity, rapidness, and selectivity for detection at very low concentrations. It can recognize all preservatives at one wavelength in less than 12 min and involves minimal sample preparation. The results of this investigation showing the presence of sodium benzoate in dairy products such as cheeses and yogurt became evident naturally, while we observed natamycin and sorbate in some samples that had conflict to authorized level of these preservatives in Iranian national standards. Therefore, it is recommended that a maximum permissible limit of preservatives should be defined for cheese and yogurt.

## Figures and Tables

**Table 1:** Analytical characteristics of the method validation.

Analyte	Calibration equation	Correlation coefficient	LOD mg/kg	LOQ mg/kg	Recovery (%)		RSDr (*n* = 3) (%)	
					Yogurt	Cheese	Yogurt	Cheese
Sodium benzoate	*y* = 81.292*x* − 15.735	0.999	0.326	0.989	94.163	93.316	±0.549	±0.799
Potassium sorbate	*y* = 41.405*x* − 20.802	0.998	0.520	1.575	88.382	85.245	±1.332	±1.394
Natamycin	*y* = 33.908*x* − 18.179	0.997	0.511	1.548	87.853	87.221	±0.549	±1.646

**Table 2:** The amount of pH, sodium benzoate, potassium sorbate, and natamycin (mg/kg) in cheese and yogurt samples.

	Brands	Preservatives (mg/kg)						pH	
		Sodium benzoate		Potassium sorbate		Natamycin			
		Mean ± SD	Range	Mean ± SD	Range	Mean ± SD	Range	Mean ± SD	Range
Yogurt	Common	18.131 ± 1.350	4.573–43.101	1.1569 ± 0.932	0–34.181	0.595 ± 0.342	0–9.640	4.23 ± .042	3.76–4.72
	Probiotic	18.122 ± 5.660	0–28.320	ND	ND	ND	ND	4.07 ± 0.12	3.79–4.49
	Vegetable	21.486 ± 2.720	0–58.188	15.601 ± 5.360	0–183.730	13.090 ± 1.798	0–66.460	4.36 ± 0.03	4.10–4.88
	Fruit	23.305 ± 4.110	8.729–40.541	178.750 ± 1.750	117.600–240.600	12.774 ± 1.800	0–27.190	4.13 ± 0.09	3.70–4.59
	Strained	10.904 ± 3.770	2.076–22.931	3.741 ± 2.426	0–11.880	4.314 ± 1.173	0–6.910	4.25 ± 0.09	3.98–4.46

Cheese	Brined	29.856 ± 4.186	3.717–125.771	12.872 ± 3.963	0–99.561	10.828 ± 5.468	0–158.025	4.79 ± 0.05	4.23–6.01
	Lactic	2.692 ± 0.275	1.735–3.457	ND	ND	ND	ND	6.63 ± 0.11	6.37–7.07
	Processed	17.322 ± 2.073	6.300–59.953	353.768 ± 70.852	0–1150.320	4.359 ± 2.385	0–62.132	6.10 ± 0.07	4.93–6.88
	Cream	13.018 ± 1.204	5.988–27.275	12.242 ± 9.867	0–247.367	1.508 ± 0.717	0–12.132	5.00 ± 0.09	4.50–6.63
	Labaneh	17.255 ± 2.403	10.241–23.617	38.679 ± 94.744	0–232.075	3.379 ± 1.575	0–8.622	5.06 ± 0.06	4.93–5.35
